# Traditional Medicine for Wound Management

**DOI:** 10.1155/2017/4214382

**Published:** 2017-07-17

**Authors:** Ipek Suntar, Satyajit D. Sarker, Lutfun Nahar, Norazah Basar

**Affiliations:** ^1^Department of Pharmacognosy, Faculty of Pharmacy, Gazi University, Etiler, 06330 Ankara, Turkey; ^2^Medicinal Chemistry and Natural Products Research Group, School of Pharmacy and Biomolecular Sciences, Liverpool John Moores University, Faculty of Science, James Parsons Building, Byrom Street, Liverpool L3 3AF, UK; ^3^Chemistry Department, Faculty of Science, Universiti Teknologi Malaysia, Johor, Malaysia

Nature is the main source of traditional medicine, which is based on the knowledge gained over generations. The development of novel drugs through the scientific investigation of biological activities and phytochemical features of traditional medicines is fundamental for the treatment of human ailments. Indeed, ethnobotanical knowledge has been recorded in folklore medicines in certain parts of the world. Ethnobotanical data are the starting point of such ethnopharmacognostic research endeavors, proceeding with an experimental part at the later stage for the verification of this information using appropriate scientific approaches.

As various natural remedies, especially from medicinal plants, are affordable and easily available, they are widely used for wound healing and to treat other skin diseases. Although the popularity of traditional and complementary medicine has increased in recent years, an awareness regarding their quality, efficacy, and safety needs to be raised through scientific standardization and safety evaluation before their clinical use.

To date, many scientific studies have revealed the wound healing active components from natural products. This special issue includes seven research articles addressing the effectiveness of natural remedies used for wound healing purposes. A research regarding the antiadhesive effect of Traditional Chinese Medicinal plants, namely,* Atractylodes macrocephala *Koidz*., Aucklandia lappa *Decne*., Cannabis sativa *L*., Citrus aurantium *L*., Codonopsis pilosula *Franch.,* Magnolia officinalis *Rehd. et Wils.,* Paeonia lactiflora *Pall*., Prunus persica *Batsch., and* Rheum palmatum *L. in intra-abdominal adhesion-induced rat model takes a part. Moreover, hemostatic activity of* Bletilla striata* Rchb.f. micron particles and potential wound healing activities of* Periplaneta americana* L.*, Abrus cantoniensis *Hance*, Prunus yedoensis *Matsum., grape seed, sesame, and fenugreek oils as well as traditionally used medicinal plants in Ghana are also presented herein. These articles represent* in vivo* and* in vitro* bioactivity tests, phytochemical analysis, and activity mechanism assays, all of which are essential for scientific confirmation of natural product utilization in complementary medicine.

We would like to express our gratitude to all authors for their contributions. We hope the readers will benefit from this special issue as an academic reference.

*Ipek Suntar* *Satyajit D. Sarker* *Lutfun Nahar* *Norazah Basar*

